# Avian Pathogenic *Escherichia coli* and *Clostridium perfringens*: Challenges in No Antibiotics Ever Broiler Production and Potential Solutions

**DOI:** 10.3390/microorganisms8101533

**Published:** 2020-10-06

**Authors:** Courtney A. Fancher, Li Zhang, Aaron S. Kiess, Pratima A. Adhikari, Thu T.N. Dinh, Anuraj T. Sukumaran

**Affiliations:** 1 Department of Poultry Science, Mississippi State University, Starkville, MS 39762, USA; caf232@msstate.edu (C.A.F.); lz245@msstate.edu (L.Z.); a.kiess@msstate.edu (A.S.K.); pa465@msstate.edu (P.A.A.); 2 Department of Animal and Dairy Sciences, Mississippi State University, Starkville, MS 39762, USA; thu.dinh@msstate.edu

**Keywords:** *Escherichia coli*, *Clostridium perfringens*, broiler, antibiotic-free, production, chicken

## Abstract

United States is the largest producer and the second largest exporter of broiler meat in the world. In the US, broiler production is largely converting to antibiotic-free programs which has caused an increase in morbidity and mortality within broiler farms. *Escherichia coli* and *Clostridium perfringens* are two important pathogenic bacteria readily found in the broiler environment and result in annual billion-dollar losses from colibacillosis, gangrenous dermatitis, and necrotic enteritis. The broiler industry is in search of non-antibiotic alternatives including novel vaccines, prebiotics, probiotics, and housing management strategies to mitigate production losses due to these diseases. This review provides an overview of the broiler industry and antibiotic free production, current challenges, and emerging research on antibiotic alternatives to reduce pathogenic microbial presence and improve bird health.

## 1. Introduction

In the United States, poultry is the leading source of animal protein, and the industry is valued at $46.3 billion from the combined production of layers, turkeys, and broilers [1]. Flock health is an increasing concern for broiler producers because diseases such as colibacillosis, necrotic enteritis, and gangrenous dermatitis result in billions of dollars in lost revenue through reduced performance and mortality [2]. In 2012 and 2013, the United States Food and Drug Administration published “Guidance for Industry #209 and #213” advising the food-producing animal industry to use antibiotics judiciously. These documents encourage the disuse of antimicrobial growth promoters as a part of a set of strategies to combat antimicrobial resistance [3,4]. In response to consumer demand, regulatory requirements, and scientific concerns, the US broiler industry has shifted most production to no antibiotics ever (NAE). The no antibiotics ever system prohibits all medically important antibiotics from being used as antimicrobial growth promoters (AGPs), which once provided a blanket of protection for broilers placed into integrator houses against bacterial pathogens such as avian pathogenic *Escherihica coli* (APEC) and *Clostridium perfringens*. In the absence of AGPs, colibacillosis, necrotic enteritis (NE), and gangrenous dermatitis (GD) have evolved as leading bacterial diseases affecting broilers raised under the NAE system.

Avian pathogenic *E. coli* causes the systemic disease colibacillosis in broilers, which is commonly characterized by the triad of lesions: perihepatitis, pericarditis, and airsacculitis resulting in septicemia and early death [5,6]. The severity of APEC disease depends on the health status of the host, virulence characteristics of the *E. coli* strain, and other predisposing factors such as stress. An estimated 30% of broiler flocks in the United States are affected by subclinical colibacillosis [5].

*C. perfringens* is a Gram-positive bacterium that causes gangrenous dermatitis and necrotic enteritis, two major diseases that cause severe economic losses to the broiler industry. Gangrenous dermatitis is primarily associated with skin lesions and subcutaneous infection and is exacerbated by environmental factors such as high litter moisture, poor litter quality, and exposure to viral infections. It is a food safety concern during processing; therefore, contaminated carcasses must be condemned or trimmed, resulting in lost revenue and increased production costs. Necrotic enteritis is a toxic infection and is characterized by hemorrhagic enteritis, high morbidity, and mortality, resulting in annual billion-dollar losses [7,8]. Necrotic enteritis can occur as primary infections as well as secondary infections in immunocompromised birds [9]. Immunosuppression by viral diseases and intestinal erosions caused by coccidia increases the risk of NE [10,11].

New challenges to broiler production within the NAE system include greater susceptibility to bacterial diseases, decreased growth performance, and higher mortality and economic losses. The objectives of this review are (1) to summarize the disease challenges and predisposing factors associated with NAE broiler production, (2) to discuss APEC and *C. perfringens* in broilers, and (3) to provide an overview of potential preventative strategies that could be employed against these diseases in NAE broiler production to reduce production losses.

## 2. “No Antibiotics Ever” Broiler Production

In 2018, broiler production accounted for $31.7 billion out of $46.3 billion in total national revenue from the poultry industry [1]. The United States poultry industry produced 8.54 billion broilers in 2014, and those numbers have continued to increase [1,12]. Over the years, poultry retail prices have remained relatively stable, making per capita broiler product consumption superior to beef and pork [13]. The success of the poultry industry in the last few decades was due largely in part to the use of AGPs in poultry feed. In 2009, 74% of medically important antibiotics sold in the US were drugs approved for use in food-producing animals as in-feed antibiotics [14]. Antimicrobial growth promoters contribute to the health and function of the broiler gastrointestinal tract (GIT), allowing producers to utilize the maximum potential of broiler genetics [15].

Previously, AGPs were added to poultry feeds to promote performance and limit disease challenges. In 2009, the Food and Drug Administration concluded that the use of antibiotics for growth promotion should be eliminated. Long term subtherapeutic antibiotic usage in food-producing animals was identified to contribute to the development and spread of bacterial resistance [16,17,18,19], which could be transmitted between humans and animals [20,21] via direct contact or through environment [22,23]. In 2014, the World Health Organization concluded that the use of antibiotics as feed additives for food animals was a public health issue as many antimicrobial agents in farm animal production were also important in treating human infections [24]. As a result, the poultry industry has come under scrutiny from medical and veterinary communities, regulatory agencies, and consumers to reduce or remove antibiotics usage in feed [25,26,27]. In 2014 and 2015, top retail customers of the broiler industry announced that they would only be serving antibiotic-free chicken [28]. Many US broiler integrators have risen to the challenge, converting most if not all of their production to NAE or reduced antibiotic-use systems. As of 2019, over 50% of birds produced in the US are under NAE programs [29]. Shifting to NAE poses a new challenge to the industry as broilers may not receive any form of antibiotics in feed, water, or injection, including the use of ionophores at any point in the chicken′s lifetime or in ovo [28].

The removal of subtherapeutic AGPs has resulted in poor flock performance, reduced daily gain, increased risk of enteric issues, low water consumption, and high mortality [22,30]. Average monthly mortality in NAE broilers is 25–50% higher than conventional broiler chickens [31]; mortality in NAE broilers averages about 4.2% compared to conventional broiler mortality at 2.9% [22]. Higher mortality in NAE couples with an increased incidence of multiple bacterial diseases, including NE and colibacillosis. Overall, NAE programs harm feed conversion ratio, body weight gain, poultry GIT health, which slows bird performance and net output [32].

## 3. Challenges in NAE Production

Previously, broiler growers depended on the administration of AGPs to maintain flock health. Antimicrobial growth promoters increased the performance and limited diseases by modifying the gut microbiota of chickens, reducing gastrointestinal inflammation, and improving the physical health of the GIT [22]. Lack of antibiotic use results in a plethora of problems including poor gut health, increased incidence of coccidiosis, increased susceptibility of broilers to environmental stressors, and a greater need to select for innate immunity during the selective breeding process.

### 3.1. Gut Health

Gut health and microbiota activity can influence broiler performance. Antimicrobial growth promoters′ primary modes of action are the modification of intestinal microbiota via reduction of opportunistic pathogens and subclinical infection, and reduction in gut wall size and villus lamina propia [25,33]. Changes in microbiota populations are beneficial to the host by altering the bacterial competition for nutrients, reducing pathogen colonization, and selecting for bacteria that can use dietary energy more effectively [25,34]. Virginiamycin when fed to Ross broiler chickens at 16 mg/kg of feed, had improved feed conversion ratio, and modified relative abundance of microbiotia within the ileum [35]. While the exact mechanism is unknown, it is suggested that AGPs provide a physiological effect on the host GIT by reducing inflammation at the intestinal mucosa, and reduction of the gut wall enhances nutrient digestibility [36]. In broiler chicken, the GIT contains the highest amount of bacterial diversity and abundance [37] with up to 10^10^ CFU/g found in the small intestine and up to 10^11^ CFU/g found in the cecum [38]. An imbalance of intestinal microflora can lead to the outgrowth and virulence gene expression of opportunistic pathogens resulting in intestinal diseases [39]. Without AGPs, broilers are at a greater risk for microbiota imbalance and diseases. Feed and feed ingredients that are contaminated with pathogenic bacteria can also introduce new pathogenic strains to broilers [40]. Nutrition and dietary composition can affect the gastrointestinal environment. For example, diets high in viscous grains such as barley, wheat, and rye increase the outbreak of NE and cause a significant reduction in performance [41,42], especially in NAE production. Changes in digesta viscosity, decreased nutrient digestibility, and prolonged intestinal transit time are possible explanations for these effects [43]. High protein rich diets, such as fishmeal, promote NE as high protein levels within the GIT act as substrates for the bacterial growth [44,45]. Understanding the gut microbial communities of broilers within NAE and making modifications is necessary to develop strategies to improve growth performance and feed efficiency, reduce intestinal diseases, and improve beneficial bacterial counts within the GIT. 

### 3.2. Coccidiosis

Coccidiosis, the most economically significant disease in poultry, is a protozoan infection of the GIT [8,46,47] and one of the biggest issues in NAE is its management. Unlike other programs, NAE in the US cannot use ionophores to control coccidiosis and relies on the use of chemical anticoccidials and vaccines [28]. Coccidiosis prevalence and infection increases the risk of many enteric diseases and is a particularly strong predisposing factor for NE [48]. Coccidia physically alters the lining of the gastrointestinal tract, change host immune status, and alter GIT microbiota. Gut damage from the protozoa increases the risk of secondary infection and peaks in subclinical coccidiosis can correspond to outbreaks of NE [28]. While chemical anticoccidials can lower the *Eimeria* load, they have no ionophore antibiotic-like positive effects on the host [49]. They are less effective than ionophores due to the rapid development of resistance, especially over prolonged periods of usage [49]. Vaccination is effective in reducing coccidiosis; however, birds vaccinated with live oocysts become susceptible to NE as live vaccines create small lesions in the epithelium of the GIT, increasing the likelihood of bacterial infection [50].

### 3.3. Housing and Environment

Environmental changes within houses can influence stress levels of birds and their susceptibility to disease. High temperatures and moisture contribute to disease pathogenicity [51]. The maximal growth rate of broilers was reported at 35 °C at 60–65% relative humidity during four to eight weeks of age [52]. However, increased relative humidity above 60% impairs heat transmission in broilers from their body core to the environment [53]. During production, increasing age and body weight increases susceptibility to heat stress [54,55]. Broilers are also susceptible to spikes in cooler weather. Su et al. noted that immunity in tracheal mucosa decreased in broilers exposed to acute 24-h cold stress from 20 °C to 7 °C [56]. Temperature stress from cold or heat, can cause oxidative stress and increase the expression levels of heat shock proteins, which can initiate an inflammatory response and decrease immune function [57]. In the NAE production system, broilers are more susceptible to these in-house environmental stressors such as litter moisture, increased ammonia levels, and heat.

#### 3.3.1. Litter and Litter Moisture

Litter is a mixture of bedding materials, spilled feed, feathers, and broiler feces [58,59]. In a single house, litter is used repeatedly for multiple flocks with the addition of plant-based bedding over the top of previously placed bedding. Reused litter increases coliform levels and coccidial outbreaks [60]. Moisture content of the litter influences the microbial activity within a broiler house [61]. Increased litter moisture is noted most often in NAE programs in the last two weeks of rearing [32]. Wet litter, litter containing 43% to 67% moisture, contained greater bacterial abundance compared to dry litter at 10% to 25% moisture content [62]. Wet litter is linked to altered digestive function, changes in feed viscosity and protein levels, increased feed passage, and is a consequence of diarrheal diseases, including both clinical and subclinical forms of NE [32,43,63,64]. It is suggested that the removal of AGPs increases the likelihood of these events. There is also an increased disease-risk as wet litter creates an imbalance in bacterial diversity of the GIT including increased amounts of Gram-positive bacteria [61,62,65,66]. Wet litter may promote the growth, survival, and transmission of *C. perfringens* and *E. coli* [67,68]. It is associated with recurrent NE outbreaks and greater prevalence of other pathogenic bacteria such as *Campylobacter* [32]. Wet litter also produces secondary health problems such as footpad dermatitis, cellulitis, gangrenous dermatitis, breast blisters, and hock burns due to ammonia proliferation [69,70,71] and can reduce overall welfare, performance, and carcass yields in broilers [72]. 

#### 3.3.2. Ammonia and Respiratory Issues

Broilers raised under NAE conditions are at 3.148 times more likely to have ammonia burns compared to broilers on an antibiotic program [73]. Broilers under NAE are at a 3.5× higher risk for developing ammonia burns of the cornea, 1.4× greater risk for foot lesions, and 1.5× higher risk of severe air sacculitis [31]. Burned feet have direct effects on bird welfare and pose infection risk as it is a site of introduction of bacteria and result in lesions that downgrade carcasses and decrease economic returns. Scratches or lacerations on the skin are portals of entry for bacteria [73] and often result in *C. perfringens* causing gangrenous dermatitis. Older birds are at increased risk of infection as they are more likely to have ammonia burns, scratches, and mouth lesions [73]. 

Exposure to broiler house dust and increased ammonia levels results in deciliation of the upper respiratory tract [74]. Inhalable dust concentrations have been reported in broiler environments at 8.29mg/m^3^ with respirable dust concentrations at 1.419mg/m^3^ [75]. Sources of dust in addition to litter includes feed, down feathers, excrement, microorganisms, and mold [76]. *E. coli* can also be isolated from the trachea, with a decrease in relative abundance overtime as broiler body weight increases [77]. Any damage to the epithelial lining of the respiratory tract such as inflammation from acute lung injury due to ammonia or heavy dust, can change local immune system environment and increases the likelihood of respiratory diseases in NAE broilers. Inflammation may alter bacterial communities present in the respiratory system and contribute to the outgrowth of opportunistic pathogens [78]. Inhalation of bacteria contaminated dust is believed to contribute to systemic APEC infections [6].

### 3.4. Selective Breeding and Lowered Immunity

Broiler growth rates have increased by more than 400% since the 1950s [79,80]. Genetic selection for faster growth rates and improved feed conversion rates has also resulted in increased infection rates [81]. There is an inverse relationship between growth rate and resistance to colibacillosis [82]. Increased infection rates are partly due to a focus on nutrient redirection and maximum growth which results in competition with the maturation of immune system and function [83,84]. For example, genetic changes selected for improving feed efficiency resulted in changes to GIT physiology and affected gut microbial population [85]. Genetic lines of chickens, including broilers, vary in their response to an *E. coli* challenge in performance traits and immune reponse [86,87]. Jang et al. discovered that Cobb broilers had greater weight loss of 64% as compared to Ross and Hubbard lines at 50% and 49% respectively when orally infected with *C. perfringens*, *Eimeria maxima*, and fed a high protein diet [88]. Cobb lines also had increased gut lesions compared to Hubbard and Ross broilers when coinfected with *Eimeria* and *C. perfringens* suggesting Cobb broilers are more susceptible to necrotic enteritis infections [88]. Yunis et al. noted that fast-growing commercial broilers had highest mortality and highest bodyweight gain, but similar antibody titer levels in response to an *E. coli* vaccine when compared to slower growing lines [82]. The current shift to NAE has instigated a need to select for broilers with a more robust immune response; it may be beneficial to select for desirable immune response and production traits [82,89]. 

## 4. Avian Pathogenic *Escherichia coli*

*E. coli* is a Gram-negative bacterium, a member of the Enterobacteriaceae family, and is aerobic and motile. *E. coli* is a natural inhabitant of the gastrointestinal microbiota of broiler chickens, their mucosal surfaces, and found readily within the poultry environment [5,38]. Majority of *E. coli* are non-pathogenic to the avian host; however, 10% to 15% of *E. coli* isolated from the GIT in broiler chickens may be pathogenic [90].

*E. coli* that cause disease within the avian host are categorized as avian athogenic *E. coli* (APEC). Avian pathogenic *E. coli* is a subset of extraintestinal pathogenic *E. coli* (ExPEC) that causes disease outside of the gastrointestinal tract. Avian pathogenic *E. coli* causes localized and systemic infections that result in production loss and cause early mortality in poultry [91]. Avian pathogenic *Escherichia coli* and human ExPEC strains share similarities in genotype, serogroups, virulence genes, and antimicrobial resistance patterns [92]. Also, APEC is viewed as a public health concern as APEC was able to cause human diseases in in vivo and in vitro models suggesting its zoonotic potential [93,94,95]. 

### 4.1. Serotypes

Serotyping APEC is essential to understanding disease prevalence and trends. Since the 1940s, serotyping has been used as a method of *E. coli* classification and uses three antigens for identification: the lipopolysaccharide (O antigen), the capsular antigen (K), and the flagellar antigen (H) [96,97]. Serotyping is an important method of classification for the ecology of isolates as it is directly associated with antigenic response [96]. Currently, 188 O groups have been established with groups O31, O47, O67, O72, O94, and O122 removed from the scheme [97,98]. APEC is linked with O1, O2, O8, O15, O18, O35, O36, O78, O88, O109, O111, and O115; with O1, O2, and O78 most correlated with APEC isolates [99,100,101,102]. Eventhough, majority of the APEC isolates belong to these specific O-serogroups, no connection linking serogroup and APEC virulence has been established [103]. A recent study conducted on the prevalene of *E. coli* within the NAE farms revealed that majority of the *E. coli* isolates with more virulence genes belonged to serogroups O8 and O78 [104]. However, more research is needed to understand these relationships. 

### 4.2. Colibacillosis

Colibacillosis caused by APEC is the most common infectious bacterial disease in poultry [105]. It is characterized by a triad of lesions of perihepatitis, air sacculitis, and pericarditis accompanied with septicemia and death [5,6]. Unlike colibacillosis in other species, colibacillosis in poultry occurs as a secondary infection when immunity is impaired. A major predisposing factor for systemic APEC infections is stress [6]. APEC strains of *E. coli* inhabit the intestinal tract and are disease-causing in the presence of stressors resulting in extraintestinal translocation [89,106]. The gas-exchange area of the lungs and airsacs are also primary routes of infection [107]. Birds are more susceptible to APEC infection and invasion due to lack of resident macrophages in their airsacs [108]. In addition to predisposing factors, virulence, and number of infectious organisms against the host′s immune response determine the duration, degree of severity, outcome, and pattern and severity of lesions [107]. Broilers in an NAE environment are more susceptible to colibacillosis and other infections due an increase in physiological stressors and lack of subtherapeutic antimicrobials [109]. 

### 4.3. Virulence Factors

Plasmids carrying virulence genes are a defining characteristic of APEC and are acquired through horizontal gene transfer [5,94]. The virulence of APEC is hard to determine as the disease often results from opportunistic infections. Variability in size, number, and virulence traits carried on plasmids exist within both APEC isolates and isolates from apparently healthy birds [110]. Certain virulence factors are shared between APEC and ExPEC strains including adhesins, toxins, protectins, iron acquisition mechanisms, and invasins that enable them to cause disease extra-intestinally [93,94]. Isolates of APEC origin may possess P-pili, S-pili, CNF toxin, Ibe proteins, or a K1 capsule, the virulence characteristics similar to human extraintestinal *E. coli* pathotypes [38]. No distinct, single virulence factor distinguishes APEC from other *E. coli*, and there is great genetic variation in colibacillosis causing APEC strains [111]. However, certain plasmid-carried virulence genes such as *hylF, ompT, iron, iss,* and *iutA* commonly occur in APEC and could be used diagnostically to distinguish APEC from non-pathogenic *E. coli* [5].

### 4.4. APEC in NAE

Risk of APEC infections in broiler flocks is influenced by various factors such as stocking density, coccidiosis prevalence, housing environment, litter quality, and viral infections [91,109,111,112]. As discussed previously, wet litter and greater ammonia levels in NAE broiler houses make the birds more susceptible to APEC infections acquired through the respiratory tract [32,73]. Moreover, lack of antibiotics in the diet might result in increased colonization of APEC strains in the broiler GIT which could eventually result in a greater risk of extraintestinal infections. There is little investigation into the fluctuations in environmental factors in commercial NAE farms and the prevalence and virulence of APEC. A recent study was conducted on the prevlance of *E. coli* within NAE farms over the course of spring and summer flock cycles. Through identification of minimal virulence predictor genes associated with APEC, *E. coli* isolates were classified for possible pathogenicity from collected samples of litter and feces, and cloacal and tracheal swabs from apparently healthy broilers. There was very high prevalence of all the five tested virulence genes (*iroN, ompT, hlyF, iss,* and *iutA*) among the *E. coli* isolates (approximately 2000 isolates) collected [104]. Moreover, a greater prevalence of samples positive for all five APEC-associated virulence genes was observed in the spring season (81.09%) than in the summer season (12.60%) [104]. This study is important as it reveals that possibly pathogenic *E. coli* exists within the NAE broilers and their environment which might result in episodes of colibacillosis outbreaks when broilers are exposed to stress.

## 5. *C. perfringens*

*C. perfringens* is a Gram-positive, spore-forming anaerobe found in many environments, including normal flora of animal and human GI tracts [113]. This anaerobe is classified under Phylum Firmicutes, Class Clostridia, Order Clostridiales, Family Clostridiaceae, and Genus *Clostridium* [113,114]. Its spore-forming ability allows *C. perfringens* to survive unfavorable conditions until it finds suitable environments [115]. Spores can be found readily in the environment of broiler chickens, which can make control difficult. *C. perfringens* causes several avian diseases, including gizzard erosions, necrotic enteritis, and gangrenous dermatitis. *C. perfringens* is a typical inhabitant of chicken microflora, but proliferates and becomes pathogenic when conditions are favorable, and a higher *C. perfringens* population density triggers expression of genes encoding toxins [8,116]. These toxins affect the gastrointestinal lining causing inflammation and deterioration of the GIT. In broilers, *Clostridium* related diseases reduce average daily feed intake by 40% and average daily gain by 16% [117].

### 5.1. Necrotic Enteritis and Gangrenous Dermatitis

It is estimated to cost the global poultry industry between $2 and $6 billion dollars every year due to NE [2,10] and was primarily controlled by use of AGPs [109]. In 2011, the prevalence of NE was as high as 30–50% for some AGP-free broiler flocks [109]. However, data on infection prevalence is scarce and may vary widely. In one commercial setting study of 51 drug-free flocks in North America, 27.4% of the flocks suffered from clinical NE, and 49.0% of the flocks suffered from subclinical NE, that resulted in increased feed conversion ratio, and decreased mean live weight at processing [32]. 

Clinical NE in poultry is characterized by a sudden increase in mortality (up to 50%) without any warning signs, and the subclinical form is associated with reduced weight gain and increased feed conversion [118]. The exact mechanism of NE pathogenesis is not well understood. Birds are infected by bacteria and spores from the environment, such as in contaminated feed, wet litter, at the hatchery, or through other affected birds [119,120]. Birds affected by NE typically have a less diverse population of *C. perfringens,* usually dominated by one or two virulent clones [121,122]. Necrotizing lesions of *C. perfringens* occur most commonly in the ileum [116]. NE affected birds appear depressed, reluctant to move, and have ruffled feathers [11,123]. Other symptoms they may exhibit include diarrhea, anorexia, and dehydration [11,124]. 

*C. perfringens* types A and C, *Clostridium septicum*, or *Staphylococcus aureus* are three of the most common agents that cause gangrenous dermatitis found in broilers either singly or in combination [125,126,127]. This disease causes skin lesions and subcutaneous soft tissue damage, and while clinical signs may not always be present, high fever, anorexia, ataxia, and later recumbency can be observed [128]. It is thought that immunsuppresion and environmental factors predispose chickens to GD [129,130]. There is an increased prevalence of GD in houses with increased litter moisture, where high incidence of skin lesions such as scratches and ammonia burns may occur [79,83]. Skin lesions associated with fighting, cannibalism, and overcrowding can serve as portals of entry for bacteria [131]. GD is commonly observed in broilers that are closer to market age (>35d) and is associated with increased condemnation rates and downgrades of carcasses at slaughter [128]. With prevalence and severity of GD increasing in the US, these downgrades will continue to increase production losses [132]. 

### 5.2. Toxinogroups and Virulence Genes

*C. perfringens* is classified into five toxinogroups (A, B, C, D, and E) based on their ability to produce major toxins; alpha (α), beta (β), beta2, epsilon (ε), and iota(ι), and the enterotoxin, CPE [113,133,134]. Other toxins produced by *C. perfringens* are referred to as minor toxins but play a critical role in the bacterium′s virulence [135]. Overall, *C. perfringens* can produce twenty toxins that play specific roles in its disease process [136]. *C. perfringens* cannot produce essential amino acids [137,138]. By using exotoxins and exoenzymes in vivo on host tissues, *C. perfringens* obtains necessary nutrients to survive [139]. 

Each set of toxins within a toxinogroup is responsible for a specific disease. In poultry, NE is caused mainly by Type A strains containing the α-toxin and the minor toxin *netB* [113]. The α-toxin is a zinc-dependent phospholipase/sphingomyelinase C and can be present in all toxinogroups [140,141]. Studies suggest that α-toxin plays a significant role in the pathogenesis of *C. perfringens* [122,142,143]. The α-toxin was thought of as the major virulence factor for NE in broilers [44,144]. However, Keyburn et al. showed that α-toxin lacking mutants of *C. perfringens* produced NE lesions to the same degree as wild type, α-toxin containing strains [145]. Moreover, *in vitro* studies of α-toxin production levels of *C. perfringens* did not correlate with the health status of chicken hosts [122,146].

Pore-forming toxin producing NetB has been proposed as the new virulence factor for NE as isolates obtained from clinically diagnosed NE broilers were positive for *netB* and produced the NetB toxin *in vitro* [147]. Isolates from NE outbreaks in US and Canada have been reported to be *netB*-positive, but *netB*-positive isolates have also been recovered from healthy broilers as well [148,149]. *C. perfringens* can be *netB*-positive but may not produce the NetB toxin [150] leaving the exact connection of *netB* and NE virulence in question. The exact mechanism of action of *netB* and NetB toxin is not well understood [147,151]. The presence or quantity of *netB* is insufficient in predicting association with virulence or pathogenicity [150,152]. 

Virulence of other genes may be associated with *C. perfringens*. The genes *netB*, *cpb2*, and *tpeL* toxin genes are found on pathogenic loci on separate large plasmids [153]. Prevalence of *cpb2*, *netB*, and *tpeL* was high in NE-producing isolates than non-NE producing isolates [152]. Genes *netB* and *tpeL* were present in human isolated *C. perfringens*, but presence may not correlate with the virulence of NE [154]. The gene *cpb2* has not been associated with virulence in broiler chickens as *cpb2*-positive isolates from diseased birds failed to produce the CPB2 toxin [150,155]. Both NE and non-NE producing isolates have been found to contain *cpb2*, suggesting that there is little association between this toxin and the disease process [135,150]. While disease producing *C. perfringens* may contain one or more once-thought-to-be virulence associated genes, the exact mechanisms of action in pathogenesis have yet to be fully elucidated. 

### 5.3. C. perfringens in NAE

*C. perfringens* in NAE broiler flocks is a concern as increased prevalence can increase the risk of disease in an already challenged system. Prevalence of *C. perfringens* was greater in drug free flocks at 13.1 strains as compared to *C. perfringens* isolated from conventional broiler flocks, averaging only 8.5 strains [32]. A major predisposing factor for NE is coccidiosis as it causes physical damage to the broiler GIT epithelium, exposes collagen, increases serum leakage, and increase mucus production into the intestinal tract; of which all can serve as nutrient sources for *C. perfringens* [11,151,156]. This problem is exacerbated as regular control of coccidia in NAE is limited. Litter moisture can also influence *C. perfringens*. It is a management concern of NAE flocks as litter moisture is often a consequence of NE due to altered digestive function [32,64] in addition to increased litter moisture in the last two weeks of rearing within NAE [32]. Increased litter moisture can increase bacterial proliferation and tends to form a microaerophilic environment more suitable for growth, survival, and transmission of *C. perfringens* [32]. It is suggested that season influences NE outbreaks with peaks occurring in late winter and early spring; however other studies have noted recurrent clinical outbreaks throughout the duration of the study suggesting strong pathogenic *C. perfringens* exists within the environment [32,109]. More insight on prevalence of NE strains is needed through monitoring of *C. perfringens* prevalence within NAE farms such as prevalence in litter content, prevalence in houses between flocks, and seasonal prevalence variations.

## 6. Disease Prevention Strategies in NAE

There is now a push in the scientific community to identify non-antibiotic alternatives that can improve bird performance and prevent the colonization of zoonotic pathogens [32]. The production shift to NAE has resulted in an increased dependence on proactive treatment of disease as opposed to a preventative treatment in conventional systems that use AGPs. Strategies to reduce disease prevalence, stress, and improve environmental and gut health are necessary to produce sustainable NAE flocks. Alternative strategies include vaccines, organic acids, essential oils, herbs, probiotics, and prebiotics, and many more. Unfortunately, none of these individual alternatives have proven as efficient as AGPs in maintaining the health status of broilers and producing as high product yields [157]. In 2015, drug-free commercial flocks were treated with a combination of non-antibiotic alternatives including an anticoccidial vaccination at the hatchery and given one of three essential-oil based feed alternatives and drinking water acidification. These flocks were compared against conventionally raised flocks. The final weights of drug-free groups were 2.06% less than conventional groups, with a decrease in FCR by six points (0.06) or 3.37% [32]. This is congruent with other research that AGP withdrawal results in decreased final body weight and increased FCR [26,109]. Broilers must be free from health challenges and placed in ideal environmental conditions to achieve full genetic potential [39]. Effective sanitation methods reduce incidence of disease [158]. Perhaps the cheapest method of disease prevention is proper implementation of biosecurity measures [159]. However, poor compliance can lead to significant disease outbreaks [160]. Strict biosecurity and cleaning protocols need to be rigorous in an NAE setting to limit sources of contamination with pathogenic microbes. NAE production will need to continue to improve alternative methods to offset the deficiencies caused by the withdrawal of AGPs. 

### 6.1. Vaccination

#### 6.1.1. APEC Vaccines

There have been many attempts to create an effective APEC vaccine (Table 1). However, APEC genetic diversity is vast, and this creates a challenge to produce an effective broad-spectrum vaccine. Early attempts in APEC vaccines resulted only in protection against homologous challenge; that is, protection was only effective against the single strain that was used to create the vaccine [152,161,162,163]. Effective vaccination whether by subunit vaccines, that target specific genetic virulence factors, or by live attenuated vaccines (LAV), have mostly resulted in coverage only against homologous challenge [164,165,166,167]. One commercially available LAV produced from nonpathogenic *E. coli* provides protection via cell-mediated immunity [168]. However, vaccinated broilers had significantly reduced weight gain compared to their unimmunized counterparts [168]. A recombinant antigen vaccine created with common ExPEC surface proteins significantly decreased bacterial loads in heart and spleen, reduced in vitro-growth of multiple APEC serotypes, and significantly decreased gross lesion scores in the air sac, heart, spleen, and liver [169]. 

Protection against APEC may be achieved through vaccination against other bacteria. Recently, Redweik et al. demonstrated white leghorns when fed probiotics and vaccinated with recombinant attenuated *Salmonella* resulted in significantly lower signs of APEC related airsacculitis, pericarditis/perihepatitis compared to the control [170]. These vaccines could decrease bacterial load of both *Salmonella* and APEC through cross-reactivity between recombinant attenuated *Salmonella* vaccine strains and APEC antigens including *iutA* and *iroN* [171,172,173].

Bacterial ghost vaccines may be an effective alternative for control against APEC. Bacterial ghosts (BGs) are bacterial envelopes of Gram-negative bacteria from the expression of cloned phiX174 gene *E*, which forms a transmembrane tunnel through the cell envelope and releases cytoplasmic contents [174]. BGs are becoming popular in vaccine development as they can produce both cellular and humoral responses [175]. A successfully modified BG APEC vaccine candidate was able to achieve over 90% immune protection of a specific serotype O2 strain with antibody levels highest in the BG immunized group [175]. BG groups also outperformed other test groups in cytokine tests and BG groups had no pathological lesions associated with colibacillosis [174]. This suggest BGs may be a new vaccine strategy for APEC prevention. Studies at commercial levels are needed to understand effectiveness in a large-scale setting. APEC vaccines once effective on a large-scale level will be a primary method to limit colibacillosis outbreaks. 

#### 6.1.2. *C. perfringens* Vaccines

Vaccination is one of the potential methods to control *C. perfringens* infections in NAE broiler farms (Table 1). Mucinases in *C. perfringens* may contribute to pathogenicity and can serve as immunogenic targets in vaccine development [176]. Toxoid vaccines of *C. perfringens* type A, C, and the combination of A and C toxoids, all resulted in a significant reduction in the number of chickens with intestinal lesions, with the most substantial lesion reduction in the A and C toxoid combination vaccine [177]. In a *C. perfringens* recombinant protein vaccination study with NetB toxin, pyruvate: ferredoxin oxidoreductase, α-toxin, or elongation factor-Tu, in combination with Montanide™ ISA 71 VG adjuvant, effects on intestinal lesion scores, body weight gain, and NetB toxin antibody levels indicated protection against co-infection of *C. perfringens* and *E. maxima* in challenged broilers [153]. Recombinant protein vaccination in combination with Montanide™ ISA 71 VG adjuvant had significantly higher weight gain, and increased antibody titers when compared to the control challenged and adjuvant alone groups [153]. A preliminary study conducted by Duff et al. identified five *C. perfringens* mucinase peptides that inhibited *C. perfringens* growth in vivo [154]. The peptides were then conjugated to an agonistic, CD40-targeting antibody and administered to live broilers challenged with *C. perfringens* and *E. maxima*. The combination of peptide vaccination improved overall performance losses and reduced lesion scores in NE-infected broilers. Vaccination can be a promising alternative tool, especially if combinations of select antigens are pooled into a single vector. Future developments in NE reduction should include anti-clostridial vaccines focused on *C. perfringens* toxin peptides and *Eimeria* antigens into a single vector [154].

#### 6.1.3. Coccidia Vaccines

NAE strategies for coccidiosis control include coccidiosis vaccination programs and hybrid vaccine-chemical strategies [28]. In 2017, 40% of US broiler integrators used coccidiosis vaccines (CV) in their programs, either incorporated as hybrids or stand-alone vaccination programs [28]. Live CV has shown to offer a protective effect for NE, reduce the severity of lesions, and lessen mortality associated with NE [10,32,155,178]. Common methods of vaccine application include intra-ocular administration, hatchery spray administration, edible gel placed on chick trays at hatchery or on feed trays, spray on feed administration, intra-yolk sac administration, and in ovo administration [179]. CV aids in flock performance as it reduces the effects of *Eimeria* or *C. perfringens* infections on weight gain [155]. The use of CVs has also decreased the incidence of gangrenous dermatitis [180]. However, live CVs induce immunity by cycling through the intestines, causing damage to the gastrointestinal tract′s epithelium, which increases the risk of bacterial disease [181]. While early exposure to oocysts challenge may boost immunity and protect chicks from later coccidiosis challenges [182], there is doubt that exposure may also compromise body weight gain and feed conversion efficiency [118]. More research is needed on the effectiveness of *C. perfringens* vaccination and NAE coccidiosis control programs and their impact in broiler performance in commercial housing separately as well as in combination settings.

### 6.2. Probiotics and Prebiotics

Probiotics are referred to as direct-fed microbials (DFM). DFMs are live microbial feed supplements that inhibit the growth of pathogenic bacteria, support growth of other beneficial microbes within the gut, and provide health benefits such as improved balance of intestinal microflora, gut barrier function, intestinal absorption, and immune status [183,184,185,186,187]. Common probiotics are *Lactobacillus* and *Bacillus* type bacteria and are clinically shown to reduce pathogens and improve performance [188] (Table 2). 

*Clostridium* and *E. coli* growth can be inhibited within the GIT by competitive exclusion with supplementation of probiotics. Probiotic *Bacillus subtilis* when added to broiler diets has been shown to lower the pathogenic bacteria counts in the GIT, improve intestinal integrity and nutrient retention, and therefore improved feed conversion [182]. *B. subtilis* 747 improved growth performance, intestinal immunity, and epithelial barrier integrity in both *E. maxima* challenged and non-challenged broilers [189]. Overall, groups of male Ross 708 broilers administered *B. subtilis* supplementation had total body weight gain averaging 553 g, comparable to AGP supplementation average of 563 g with all groups outperforming the challenged control (493 g) [189]. Ramlucken et al., when testing a selective multi-strain *Bacillus* probiotic mixture of *B. subtilis* CPB 011, CPB 029, HP 1.6, and D 014, and *B. velezensis* CBP 020 and CPB 035, noted improved feed conversion ratio, increased body weight gain, and overall improved performance compared to unsupplemented and commercial *Bacillus* supplemented flocks when challenged with *C. perfringens* [190]. Probiotics may also promote economic savings of $0.018 USD/kg of body weight when *B. subtilis* was included in a two percent reduced metabolizable energy diet [191]. Multi-strain probiotics of *Lactobacillus acidophilus*, *B. subtilis*, and *C. butyricum* have shown to improve chickens′ gut health. This combination probiotic improved the ileal absorption of most essential amino acids, increased *Lactobacillus*, reduced *E. coli* counts in the GIT, and reduced NH_3_ in excreta odor content [192]. Combining probiotics with other prevention strategies also has shown promise in reducing performance losses. Probiotic administered in drinking water along-side coccidiosis vaccination may reduce the effects of coccidiosis vaccination on chick growth [193]. *B. subtilis* fed in combination with prebiotic mannan-oligosaccharides and beta-glucans to broilers exhibited higher body weight gain overall from d0 to d41 (*p* < 0.039); however, treatments did not affect *E. coli* levels within the ileum at any age [194]. Finding the right combination of probiotic with other prevention strategies could reduce GIT lesions caused by *C. perfringens*, reduce incidence of pathogenic *E. coli*, and improve overall bird health and performance. 

An additional alternative to include in broiler production is the use of prebiotics (Table 2). Prebiotics are substances that promote intestinal microbial growth and overall host health [195]. Prebiotics are not digested by the broilers and promote the growth of beneficial bacteria and improve flock performance. Common prebiotics help prevent pathogens from infecting the host by blocking binding sites on the intestinal epithelium; this includes nondigestible oligosaccharides such as mannooligosaccharides [196] and isomaltooligosaccharide [197]. Prebiotics can be used in combination with *B. subtilis* probiotics in broilers without compromising feed conversion ability [181]. The addition of mannooligosaccharides in diets of broilers challenged with *E. tenella* had significantly reduced lesion scores (0.29) compared to lesion scores of the challenged negative control (2.93) [197]. In some instances the mannooligosaccharide added diets outperformed amprolium hydrochloride treated chickens in significant reduction of mucoid contents in cecum, as well as a reduction in bloody fecal contents [197]. Isomaltooligosaccharide, when fed to commercial broilers challenged with APEC, improved growth performance, and modulated intestinal microbiota by increasing *Lactobacillus* numbers [198]. Prebiotics and probiotics are beneficial alternatives in promoting gut microbial balance, gastrointestinal health, and performance benefits in weight gain and feed conversion ratio. When used in combination with proper management, vaccination, and other strategies to reduce disease risk factors, prebiotics and probiotics can provide multi-factorial benefits to the broiler industry. 

### 6.3. Biosecurity

The health of NAE broilers depends on many factors associated with the early phases of broiler production, which include the breeder facilities and the hatcheries. Quality chicks begin with clean hatching eggs, proper hatchery sanitation, hatchery management, and brooder management, and NAE farms must ensure health guarantees from the hatchery on day-old chicks. Kim and Kim reported that operating hatchers pose a large contamination risk with aerobic bacteria, coliforms, and fungi as high as 300 CFU/63.6 cm^2^ [207]. To maximize hatchability and chick quality, it is important to reduce the microbial prevalence on eggshells [208]. Reduction of microbial contamination on eggshells has been successful with use of essential oils [209,210,211]. Clove essential oil, *Syzygium aromaticum*, has been shown as a promising spray antimicrobial alternative in microbial load reduction off eggshells [209]. All hatchery equipment should be inspected and have regular cleaning, sanitizing, and maintenance schedules to ensure top quality chicks. To establish the prevalence of various bacteria and to identify sources of contamination, routine swabs and cultures should be taken on chick-contact surfaces along with regular monitoring of air ventilation, temperature, and humidity. 

There are internal and external factors affecting the biosecurity of NAE farms such as disease management, cleaning, and sanitation and hatchery chicks, and monitoring visitors and personnel on the property. NAE broiler production requires strong biosecurity measures including restricted outside human contact with flocks. Pest control should also be closely monitored as wild birds, rodents, and parasites can carry disease and produce detrimental effects on production performance. Daily management of flocks should include removing carcasses from houses, conducting post-mortem examinations to monitor disease presence, and disposing of diseased birds away from the immediate housing environment. 

### 6.4. Housing and Environment Management

Control begins with cleanliness and management practices in both breeder and broiler houses. Lighting, ventilation, litter quality, spacing, and pest control must be rigorously managed. Feed and water line contamination should be tested and treated as needed. Litter quality is correlated with moisture levels and is impacted by the number of broilers in a flock [212]; therefore, litter amendments, changing litter, or windrowing may be included in NAE production to reduce microbial load. A recent study suggests stocking density influences litter moisture [213]. While commercial NAE settings revealed no significant difference in feed conversion, mortality, or body weight gain, a low stocking density (0.27 m^2^) showed a 2.5% reduction in litter moisture when compared to the higher stocking density (0.23 m^2^) [213]. Litter moisture has been associated with increased ammonia levels, increased bacterial load, and increased lesions such as footpad dermatitis, breast blisters, and hock burns [70,71,72]. Zuowei et al. reported that broilers raised in lower stocking densities had higher BW and lower FCR [214]. Reducing stocking densities may mitigate litter moisture, lower housing temperatures, and decrease infection risk. However, lower stocking densities than industry standards did not show any significance in feed conversion, mortality, or BW [213]. Winkler et al. found that litter moisture was highest around water lines at 40.7% in tandem with the increased prevalence of *E. coli* and *C. perfringens* in the same locations [215]. Areas of higher litter moisture will also contain higher levels of fecal related microorganisms.

Downtime between flocks decreases the prevalence of pathogens and increasing downtime between flocks by an additional seven days, compared to the minimum of seven days [216] has resulted in a 50% return on costs, primarily due to the reduction in coccidiosis challenge [28]. The result is that NAE producers have increased downtime, averaging 16 days between flocks [28]. In addition to current strategies of cleanliness and biosecurity measures, novel and non-conforming methods are becoming more commonplace in the poultry industry to combat pathogenic disease and improve NAE broiler performance.

### 6.5. Other Methods

Numerous studies have shown the antibacterial effects of various essential oils (EOs), including thymol, carvacrol, eugenol, rosemary, oregano, geraniol, cinnamaldehyde, and curcumin [217,218,219,220,221]. For *C. perfringens*, EOs added to feed reduced *C. perfringens* intestinal counts, lesion severity, and mortality associated with NE [219]. Most studies on EOs and their effect on *C. perfringens* is against a challenge rather than an established clinical disease, and this may not be an accurate representation of commercial field effectiveness [219,222]. Alternative EO based products can be used to treat clinical NE outbreaks; however, the products did not control NE in the field as efficiently, economically or as rapidly as AGPs [219,222,223,224,225]. Treatment of infected flocks was slower with the EO based product and affected daily weight gain, FCR, and final weights of AGP-free flocks [39]. EO based feed products have been shown to inhibit *E. coli* in vitro [220,226] and within the lower intestinal tract of chickens [227].

Organic acids have been shown to affect *C. perfringens* growth without affecting the intestinal micro-architecture of the poultry GIT and may contribute to mitigating NE in NAE flocks [228,229,230]. It is suggested that supplementation of organic acid in the drinking water lowers pH, improves antibacterial effect, and improves water quality [228,229,231]. The use of organic acids has been shown to decrease the prevalence of pathogenic bacteria such as *Salmonella*, *Campylobacter*, and *E. coli* [232], and when added to drinking water, it helped regulate gut microflora and increase digestion of feed [233]. Table 3 provides a list of current alternative methods of other categories that may prove beneficial to broiler production. Producers should keep in mind the stocking density of flocks and its effect on microbial growth, stress, and effects on ammonia and litter moisture that may proliferate pathogenic bacteria.

## 7. Conclusions

The broiler industry is a major sector of animal agriculture in the United States and continues to expand. Broiler flocks are at an increased risk for infection and mortality due to major integrators transitioning to antibiotic free programs. Fast-growing broilers are no longer supplemented with subtherapeutic levels of antibiotics or AGPs, that once protected broilers from major infections. Challenges within NAE broiler production include poor gut health, greater coccidiosis prevalence, adverse litter conditions, stocking density, and respiratory issues. Without the use of AGPs, morbidity and mortality rates have increased within NAE farms. The most common bacterial diseases associated with these losses are colibacillosis caused by APEC and necrotic enteritis and gangrenous dermatitis caused by *C. perfringens.*

Producers are searching for novel antibiotic alternatives to improve flock health and mitigate the increased morbidity and mortality rates seen in antibiotic-free broiler production. Vaccination for *E. coli* and *C. perfringens* are showing promising results especially in combination with prebiotic and probiotics. However, more studies are needed in large scale commercial settings to fully elucidate the effectiveness of novel vaccines. Prebiotics and probiotics such as *Lactobacillus and B. subtilis* are common additives now in poultry feed to reduce GIT inflammation, promote intestinal absorption, and improve microbial flora. Stocking density also influences litter moisture, stress level in broilers, and microbial load; all factors that contribute to the health status of the flock in NAE environments. Efforts to reduce pathogenic microbial load within NAE can be achieved by having clean and sanitary environments in all sectors of vertical integration beginning with the hatchery. Ensuring prime cleanliness, sanitation procedures, and strict biosecurity are the most cost effective methods to reduce disease risk. No single antibiotic alternative is as effective in a stand-alone challenge against conventional AGP methods. Through combination strategies such as stringent biosecurity measures, routine vaccinations, in-feed prebiotic and probiotics, essential oils, and organic acids, producers can better manage NAE flock health and reduce incidence of common ailments from pathogenic *E. coli* and *C. perfringens*.

## Figures and Tables

**Table 1:** Reports on the efficacy of various vaccines against APEC and *C. perfringens* in broilers.

Target Microbe	Vaccination and Results	Reference
APEC	Purified outer membrane vesicle (OMV) proteins derived from APEC serotype O78 given in vaccination to challenged Lohmann chickens showed protection over non-vaccinated groups. Native APEC O78 OMVs provided protective immunity in chickens challenged against corresponding serotype bacteria.	[183]
APEC	Male and female white leghorns vaccinated with recombinant antigens of common ExPEC surface proteins and then challenged with APEC had significant IgY response, reduced in vitro growth of multiple APEC serotypes, decreased internal bacterial loads and reductions in gross lesion scores in airsacs, heart, liver, and spleen	[169]
APEC	Bacterial ghost vaccine of APEC O2 isolate was able to achieve over 90% immunity in challenge broilers and high antibody response of 120 Sanhuang broiler chickens.	[174]
*C. perfringens*	Combination vaccine of 5 mucinase peptides of *C. perfringens* showed promise in improving BWG in subclinical NE challenged broilers.	[154]
*C. perfringens*	*C. perfringens* toxoid vaccination of A, C, and combined A and C toxoids in broilers resulted in decreased intestinal lesions, and increased antibody titers, especially after the second booster dose.	[177]
*C. perfringens*	*C. perfringens* recombinant protein vaccination with NetB toxin or pyruvate: ferredoxin oxidoreductase in combination with Montanide™ ISA 71 VG adjuvant had significantly higher weight gain, and increased antibody titers than control challenged and adjuvant alone groups in broilers challenged with oral co-infection of *C perfringens* and *E. maxima*.	[153]

**Table 2:** Reports on the efficacy of probiotics and prebiotics against APEC and *C. perfringens* in broilers.

Strategy	Results	Reference
Probiotic	*B. subtilis* strain 747 improved growth performance, intestinal immunity, and epithelial barrier integrity of broiler chickens	[189]
Probiotic	A multi-strain *Bacillus* probiotic, 4 *B. subtilis* (CPB 011, CPB 029, HP 1.6, and D 014) and 2 *B. velezensis* (CBP 020 and CPB 035), improved growth performance and improved gut and liver function of broilers when under challenge.	[190]
Probiotic	*B. subtilis* DSM 32315 controlled proliferation of *C. perfringens* in intestines of broilers under challenge, reduced performance loss and partially replaced in-feed AGP.	[39]
Probiotic	Feed supplementation with *L. johnsonii* BS15 in the prevention of subclinical NE in broilers was effective in influencing performance (higher ADG and lower FCR) when given before NE challenge. BS15 effects were limited in groups with established development of NE.	[199]
Probiotic	Broiler groups under *C. perfringens*, *Eimeria* challenge, and fishmeal supplementation when fed *B. licheniformis* had similar cecal microbiota compared to that of the control group, suggesting that *B. licheniformis* disrupts microbiota and alleviates cecal disruption caused by multiple gastrointestinal challenges.	[200]
Probiotic	Broilers challenged with *E. coli* K88 and fed *L. plantarum* B1 had increased BW, decreased *E. coli* counts, and increased lactic acid bacteria in the ceca compared to challenged untreated counterparts. Broilers fed *L. plantarum* increased ileal mucosal secretory IgA and reduced IL-2, IL-4, IFN-γ, and tumor necrosis factor-α levels in the ileum.	[201]
Probiotic	Broilers fed *L. plantarum* during the entire growing period or finishing period (d22-42) performed better overall than broilers fed only in starter period or no supplementation.	[202]
Probiotic	A multi-strain probiotic containing *L. acidophilus*, *B. subtilis*, and *C. butyricum* improved FCR, ileal digestibility, increased *Lactobacillus* and decreased *E. coli* in the GIT, and reduced NH_3_ excreta content compared to control broiler groups.	[192]
Prebiotics, Probiotics, and combination	Broilers fed *B. subtilis* spores, or combination of commercial prebiotic, Mannan oligosaccharide, and *B. subtilis* spores exhibited overall higher BW gain compared to negative control and AGP positive control diets.	[194]
Prebiotic and Probiotic	The prebiotic and probiotic combination improved digestive organ growth of broilers, but did not improve growth or meat yield of broilers	[182]
Prebiotic	Sodium butyrate (Na-B) significantly lowered intestinal lesion scores compared to control challenged Cobb-Cobb male broilers.	[203]
Prebiotic	Broilers fed sweet orange peel extract levels in concentrations higher than 1000 ppm improved rates of IBD and IBV antibody titers and immune response in broiler chickens	[204]
Prebiotic	Use of quercetin, a ubiquitous flavonoid, altered cecal microflora of broilers by reducing *P. aeruginosa, S. enterica*, *S. aureus*, and *E. coli*, but increased copies of *Lactobacillus* and Bifidobacterium; inhibited growth of *E. coli* and *S. aureus* in vitro by damaging cell wall and cell membrane structures; and had bactericidal effects on Gram-positive bacteria	[205]
Prebiotic	Isomaltooligosaccharide improved hot carcass weight and increased *Lactobacillus* microbial numbers in the ceca with broilers under challenge from *E.coli* O78 (APEC)	[198]
Prebiotic	Broiler groups fed 300mg/kg *Beta vulgaris* extract had comparable FCR to anticoccidial treated groups. *B.vulgaris* extract improved FCR, reduced oocysts in feces and lesion scores in *Eimeria* sp. challenged groups	[206]
Prebiotic	Ross male broilers treated with mananoligosacharide when challenged with *E. tenella* significantly outperformed control and treated groups with amprolium hydrochloride with improved FCR, body weight gain, and feed intake.	[197]

**Table 3:** Other preventative strategies and antibiotic alternative effects in broiler production.

Strategy	Results	Reference
Competitive exclusion	Commercially available competitive exclusion culture administered via oral infection to White Leghorn chickens on day 1 of placement reduced the number of ESBL/AmpC-producing *E. coli* in gut cecal contents	[234]
Environment	Reduced stocking density in NAE broiler flocks decreases litter moisture	[213]
Environment	Reduced stocking densities of broilers had higher BW and lowered FCR than high stocking densities	[214]
Environment	Broilers raised at the lower stocking density had higher BW, but lower FCR	[235]
Genetics	No difference in AMR presence of *E. coli* in fast-growing vs. slow-growing breeds of broilers in an antibiotic-free system	[236]
Organic Acids	A meta-analysis of 121 articles on organic acids in broilers showed that organic acids blends were most effective in increasing ADG and FCR compared to organic acids used alone. Birds under challenge were positively affected in FCR when organic acids were used but not to the same extent of AGPs	[237]
Essential Oil	An in vitro study of screening 28 different essential oils revealed potential selective antibacterial activity of *E. globulus*, *E. exserta*, *P. pseudocaryophyllus*, Orange Oil Phase Essence, and Citrus Terpenes oils against pathogenic bacteria and little antibacterial activity observed in beneficial microbes such as *L. plantarum* and *L. rhamnosus*	[238]
Essential Oil	Total aerobic mesophilic bacteria prevalence was significantly lower (2.30 log10 CFU/mL) in clove essential oil sprayed eggs than nonsanitized eggs (3.49 log10 CFU/mL) comparable to traditional sanitizer, paraformaldehyde (2.23 log10 CFU/mL).	[209]
